# Uncovering the extent of dementia prevalence in Iran: a comprehensive systematic review and meta-analysis

**DOI:** 10.1186/s12889-024-18415-y

**Published:** 2024-04-25

**Authors:** Sima Oshnouei, Mahin Safaralizade, Nazila Farrokh Eslamlou, Mohammad Heidari

**Affiliations:** 1 Phd Candidate of Epidemiology, School of Public Health Iran, University of Medical Sciences, Tehran, Iran; 2 Phd Candidate of Information Science & Knowledge - Data Recovery, Urmia University of Medical Sciences, Urmia, Iran; 3 Phd of English Language Teaching, School of Medicine, Urmia University of Medical Sciences, Urmia, Iran; 4 Phd of Epidemiology, Social Determinants of Health Research Center, Clinical Research Institute, Urmia University of Medical Sciences, Urmia, Iran

**Keywords:** Alzheimer, Dementia, Prevalence, Iran, Systematic review, Meta-analysis

## Abstract

**Background:**

Dementia is one of the major causes of disability and dependency among older people worldwide. The formation of an aging population in Iran can be associated with societal problems, including age-related disorders such as dementia. This study aimed to estimate the prevalence of dementia& Alzheimer disease in adults aged 60 years or older and it’s its geographical distribution in Iran.

**Methods:**

A systematic review and meta-analysis study included articles published in both English and Persian languages and utilized various databases including: Google Scholar, PubMed, Web of Science, Magiran, and thesis database of medicine universities up to December 2022. The pooled prevalence was calculated using random effects models. The prevalence was reported separately for different geographical locations and types of area sampling, and age adjustment was performed for the selected studies. All statistical analyses were conducted using metaprop package in STATA version 17. The I2 statistic was applied to assess heterogeneity.

**Results:**

The meta-analysis considered nine relevant studies that were carried out up to 2023 in Iran. The study found that the prevalence of dementia in central and east counties was estimated to be 0.14 (95% CI; 0.04–0.31), while in western counties, the prevalence was estimated to be 0.1 (95%CI; 0.01–0.27). The estimated overall crude prevalence of dementia was estimated at 0.14 (95% CI; 0.03–0.31). Estimated prevalence-based health centers sampling and hospital-based studies were 0.02 (95% CI; 0.02–0.03), 0.05 (95% CI 0.06–0.11), respectively. One study used nursing home sampling as the sampling method, and the estimated prevalence was 0.43 (95%CI 0.38–0.49).

**Conclusion:**

This is the first systematic review and meta-analysis of the prevalence of dementia’s disease up to 2023 in Iran. The estimated overall prevalence of dementia is lower than the reported prevalence in European countries and similar to other Asian countries.

**Supplementary Information:**

The online version contains supplementary material available at 10.1186/s12889-024-18415-y.

## Introduction

Dementia is one of the major causes of disability and dependency among older people worldwide. It is an umbrella term that encompasses several diseases that affectmemory; Alzheimer’s disease (AD) being the most common form accounting for 60–70% of cases. The total number of individuals affected by dementia is projected to increase to 82 million in 2030 and 152 million in 2050. This growth is expected to be more pronounced in lower- and middle-income countries compared to high-income countries [1]. The World Health Organization predicts that by 2050, approximately 1.2 billion people will be aged 60 years or older, with around three-quarters of them residing in developing countries that are considered to have a higher risk of dementia [2].

The rapid demographic transition in Iran has led to the formation of an aging population, which can be associated with adverse health issues, including a surge in age-related disorders such as dementia. According to Global Burden of Disease (GBD) study, during the years 1990 and 2017, the incidence rate of AD in Iran was 75.81 per 100,000 (62,298 persons) for all age groups [3].A national study reported that the disability related to AD, as measured by the age-standardized YLD rate, decreased from 171 to 220 per 100,000 persons in 1990 to 133–220 per 100,000 persons in 2015 [4]. Despite this, the increasing prevalence of dementia in Iran and worldwide is alarming, necessitating the implementation of new strategies to address the growing demand. A population-based study, conducted in Tehran, reported a prevalence of mental impairment of 1.7% in 2011 [5].

In 2017, the per-person cost of AD in Iran was 434 USD, 1313 USD, and 2480 USD for mild, moderate, and severe stages of AD, respectively. Additionally, a study conducted in Tehran City between 2016 and 2017 reported a yearly economic burden of 810,391,868 US dollars for AD. The total cost spent for each patient was 683.9 dollars (including 35,000 rials in 2015) [6]. A recent Systematic Review estimated the prevalence of AD in Iran to be 2.3% up to 2017, without the use of meta-analytic strategies [7]. Previous studies on Alzheimer/dementia conducted around the world, particullarly in European countries, have highlighted the impact of age, gender, diagnostic criteria, and living areas of patients and social/cultural norms on the prevalence of the disease and the diagnosis and treatment of dementia patients [8–10].

Considering the geographical variation and different levels of risk factors and the increasing trend of the ageing population [11], this Systematic Review and meta-analysis aimed to evaluate all relevant documents pertaining to the prevalence of dementia and Alzheimer’s disease in Iran. The study sought to estimate the pooled measure and investigate potential causes of heterogeneity in the prevalence rates.

## Methods

The protocol of this systematic review and Meta-analysis has been registered in PROSPERO with ID: CRD42020167630 and was approved by the Ethical Committee of Urmia University of Medical Sciences (No: IR.UMSU.REC.1398.385).

### Study inclusion criteria

Inclusion Criteria: The inclusion criteria for this study are as follows:

1. The title, abstract, or keywords of the articles should mention the specified keywords.
2. Articles written in both Persian and English languages are eligible for inclusion.
3. The articles should provide information on the age of participants, including Mean (SD), as well as the prevalence and incidence of Dementia and Alzheimer’s. Studies focusing on the treatment of Dementia and Alzheimer’s, genetic epidemiology, will be excluded.addingly Studies with an inappropriate study design (e.g., case reports, qualitative studies), Studies with outcomes not relevant to dementia prevalence and high risk of bias or poor methodological quality or focus on interventions rather than prevalence rates. Given the limited amount of original research on the prevalence/incidence of dementia and Alzheimer’s in Iran, we aimed to increase the search sensitivity. As a result, reviewers independently evaluated the titles, abstracts, and other publications of all identified articles. Only articles that met the following criteria were included in this study: population-based random sampling, surveys, epidemiologic studies estimating the incidence, prevalence, or frequency of dementia or Alzheimer’s; studies reporting years of life lost due to dementia or Alzheimer’s; studies reporting the direct and indirect costs of this disease in Iran; and other systematic reviews conducted worldwide and in the Middle Eastern region (if available), which met high-quality standards.

### Data extraction and management

Two investigators (OSH S, S M) conducted literature searches and extracted data, ensuring two independent evaluations for each record. Disagreements were resolved based on the scientific opinion of H M.

Data Extraction: The following data items were collected from each article: Authors, Titles, Year of sampling, Type of sampling or methods used for population recruitment and data collection.

Geographical area, Demographic characteristics (age, sex, living area), Diagnostic criteria for Alzheimer’s or dementia, Number of participants in the study, Direct and indirect costs of the disease, Years of life lost due to the disease, Prevalence of the disease.

### Search strategy and study selection

In this systematic review and meta-analysis study, we conducted a comprehensive search in both Persian and English-language databases. The databases searched included Medline, Google Scholar, PubMed, Web of Science, Magiran, and the thesis database of medicine universities. The search was performed using the MeSH terms: prevalence, frequency, epidemiology, Alzheimer’s, dementia, and Iran. No time limit was imposed, and the search encompassed articles published up to 2023. Furthermore, we extended our search to include other sources in the Persian (Farsi) language. These sources included abstracts, conference proceedings, titles of theses, dissertations, and reports from databases such as IranMedex, IranDoc, Iranian Archive for Scientific Documents Center (IASD), as well as the websites of Iran alzheimer’s and dementia association and the Iranian national library (INL). To ensure a comprehensive search, we also manually examined the references of selected citations. In this process, we considered equivalent persian keywords for their corresponding english words and explored all possible combinations. PRISMA flow diagram was used to depict the flow of information through the different phases of a systematic review and meta-analysis.

### Risk of bias (quality) assessment

Two reviewers (MH and SO) independently assessed the risk of bias in all selected studies and collaborateed to reach a consensus on their assessments. To evaluate the quality of non-randomized studies, we employed a scoring system based on the modified Newcastle Ottawa Scale (NOS) adapted for cross sectional studies. This scoring system considered various factors in three main dimain including Selection (Maximum 5 stars), Comparability (Maximum 2 stars), Outcome (Maximum 3 stars) respectively. Subdomains were Methodological quality (risk of bias) issues including: research design appropriateness, recruitment strategy, response rate, representativeness of the sample, objectivity/reliability of outcome determination, provision of power calculation, and utilization of appropriate statistical analyses. To reach a consensus, disagreements in scores were resolved through discussion, and a final rating was assigned to each study.

### Data analysis

The study extracted data using microsoft excel and analyzed it using Stata™ version 17.0. (STATA Corporation, College Station, TX, USA). The metaprop package was utilized to derive weighted average prevalence estimates. Overall estimates were calculated using random effects model the method of DerSimonian and Laird, and heterogeneity was assessed using chi-square and I2 statistics. To stabilize the variances we used Freeman-Tukey Double Arcsine Transformation (Freeman, M. F., and Tukey, J. W. 1950) for pooled estimate. The cimethod parameter, which specifies a different method, was used to construct the score (Wilson) confidence intervals for each study. Conducting a straightforward sensitivity analysis means recalculating the overall effect size by leaving out the first study that was published. Furthermore, to pinpoint whether a particular study has an unusually significant impact on the overall estimate, we recalculate the combined effect size while excluding each study one by one through a “jack-knife” procedure.We carry out this process in STATA using the metaninf command.

### Subgroup analyses

To identify the source of heterogeneity, subgroup analysis, publication bias, and sensitivity analysis were computed. A two subgroup analyses were desihned based on population sampling (health centers sampling/ Hospital-based studies/ nursing home sampling), geographical Location (central and east / west counties).

## Results

### Characteristics of included studies

During the initial screening with a higher sensitivity vs. specificity approach in the database search, we identified 100 records published between the years 2002 and 2023 that focused on various aspects of dementia and alzheimer’s disease in Iran. The eligibility of articles was assessed, and full texts were considered for systematic review and meta-analysis.

**Fig. 1:**
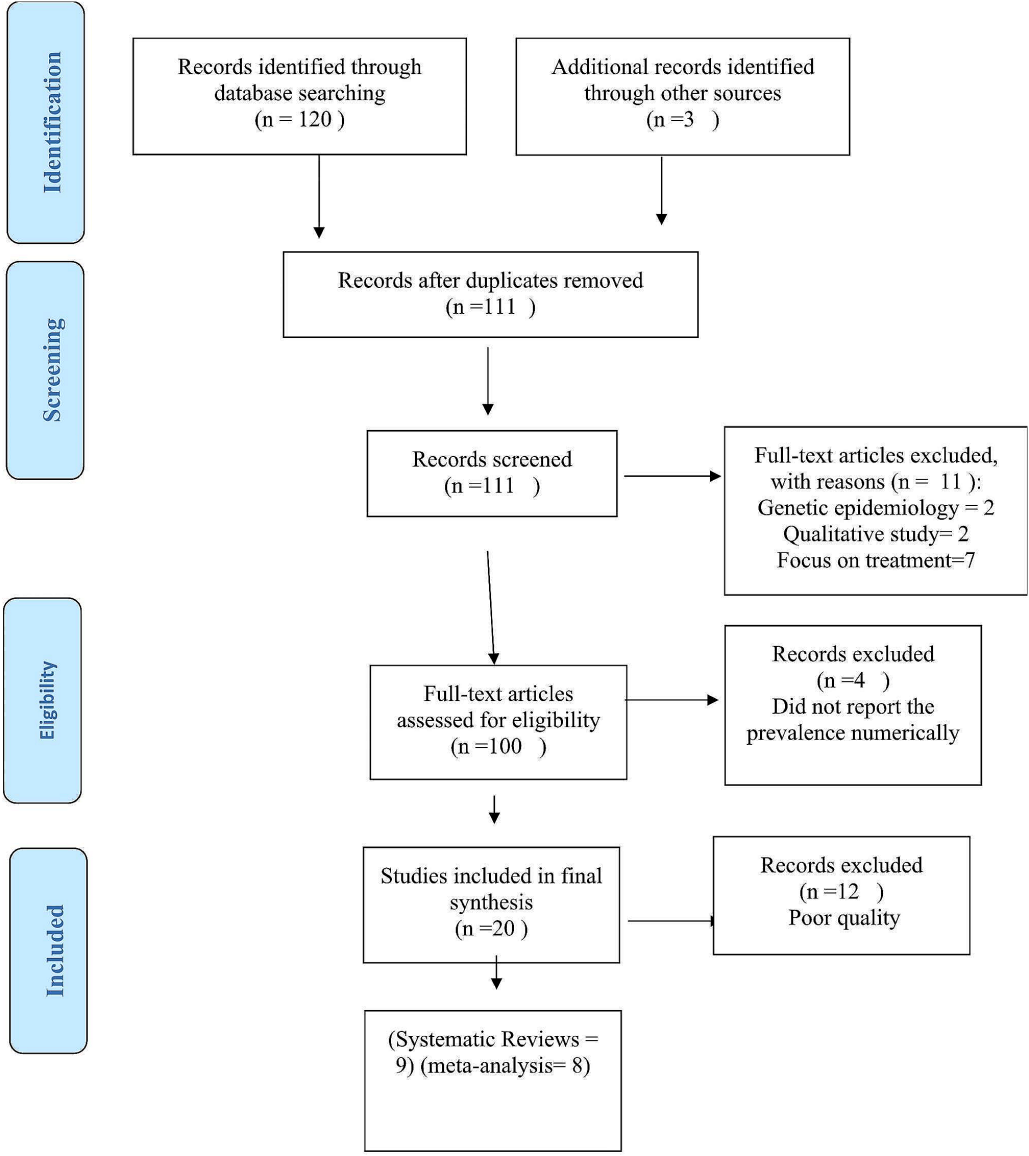
: PRISMA flow diagram of studies included and excluded at each stage of screening. PRISMA, preferred reporting items for systematic reviews and meta-analyses

Table 1 provides an overview of the main characteristics of the 10 articles included, which reported the prevalence and frequency of dementia and alzheimer’s disease. The results of the Newcastle-Ottawa Scale (NOS) were shown in Table 1, illustrating the criteria for allocating stars to assess the quality of studies. All data generated or analyzed during this study are included in this resuls. Figure 1 presents the PRISMA flow diagram depicting the studies included and excluded at each stage of the screening process.

**Table 1:** Characteristics of the included studies in this systematic Review and meta analyses

Author	Published year	City	Age range	Studied Population	Diagnostic Criteria	No of Alzheimer /dementia patience	NOS *			
							Selection	Comparability	Outcome	
Sadegi M	2004	Tehran	65 years ≤	279 Convenience Sampling, 26 center nursing home	DSM-IV	121	**	*	***	
Mohammadi A [12]	2012	Boyerahmad	65 years ≤	804 Stratified three stage cluster sampling	Persian version of Clinical Dementia Rating Scale (CDR) Moderate and Sever Dementia were included)	85	***	*	***	
Sharifi F	2016	Alborz	60-80years ≤	227 Multi-stratified cluster proportionate random sampling method	Brief Cognitive Assessment Tool (BCAT) In briefly: the impairment in the three-word recall test as well as the inability in one of the mentioned domains of ADLs.	15	*****	**	***	
Sharifi F	2016	E. zerbaijan	60-80years ≤	403 Multi-stratified cluster proportionate random sampling method	//	20	*****	**	***	
Sharifi F	2016	Khuzestan	60-80years ≤	353 Multi-stratified cluster proportionate random sampling method	//	34	*****	**	***	
Sharifi F	2016	N.Khorasan	60-80years ≤	96 Multi-stratified cluster proportionate random sampling method	//	14	*****	**	***	
Sharifi F	2016	S. & Balouchestan	60-80years ≤	178 Multi-stratified cluster proportionate random sampling method	//	19	*****	**	***	
Gholamzadeh S	2017	Shiraz	60 years ≤	2000 Random sample health center	Mini-mental state exam(MMSE)/geriatric depression scales (GDS)	46	*****	**	***	
Kamalzadeh L	2019	Tehran	60 years ≤	205 Hospital based sampling	DSM-5	45	***	**		***
Davarinejad O	2019	Kermanshah	< 25–50≤	275 Hospital base random sampling	-	5	***	**		***
GBD DC ^	2019	World / part of it is about Iran	60 years ≤	4,361,843 Tehran&Babol Universities of Medical Sciences	DSM; DSM-III, DSM-IV, or DSM-5)	368,528	***	**		***
Hosseinzadeh A	2022	Iran	60 years ≤	Indirect Estimation of Dementia Prevalence and Its Geographical Variation Using the Claim Data in Iran	The frequency of prescribed specialized medicines in one year by generic and brand names in each province	508.9 per 100,000	***	**		***
BBC Report 2019 [13]	2018	Iran	60 years ≤	General Population	-	700,000	-	-		-

* Newcastle-Ottawa Scale (NOS) Illustrating the Criteria for Allocating Stars to Assess the Quality of Studies

^ GBD 2016 Dementia Collaborators

### Prevalence of dementia

**Fig. 2:**
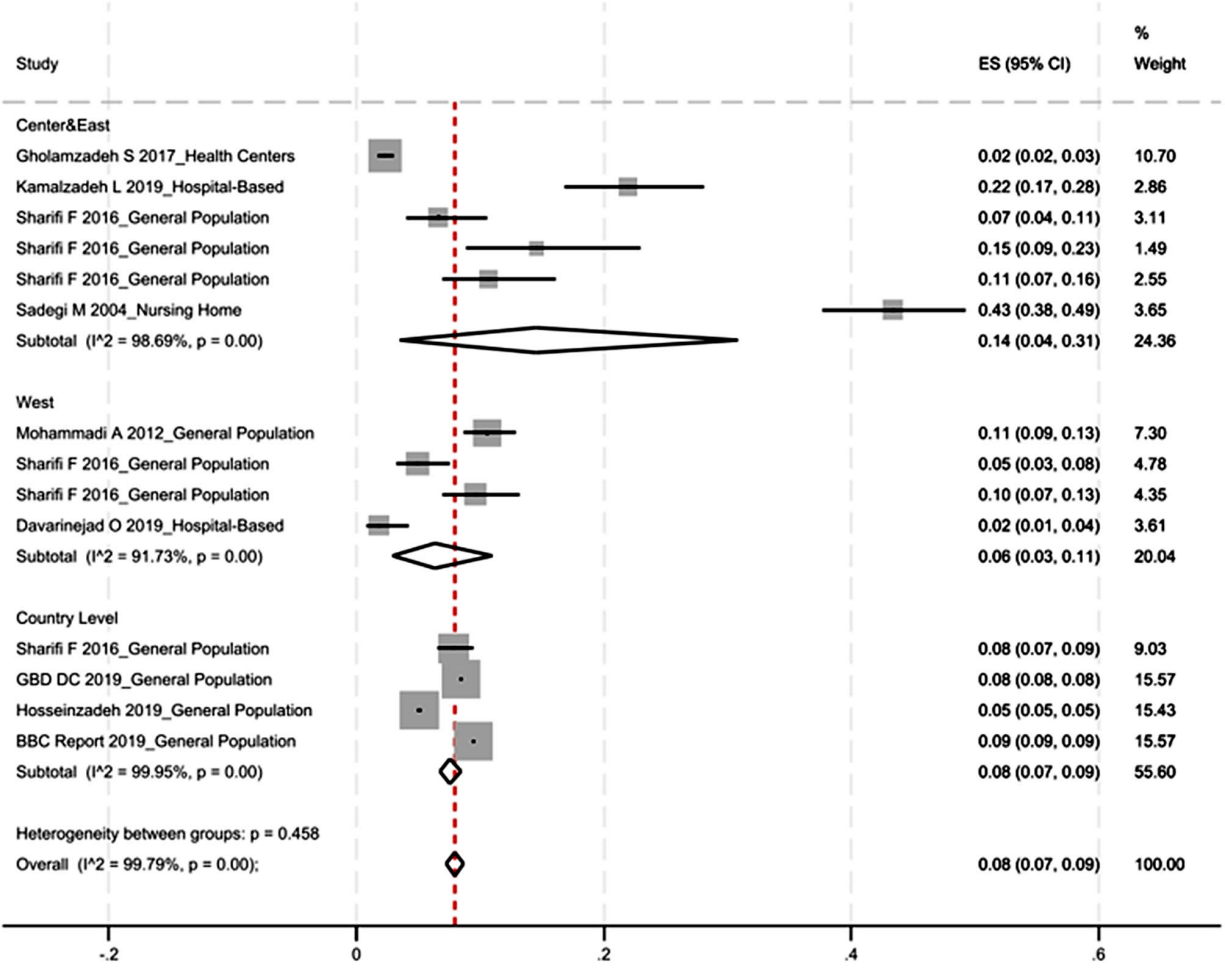
The crude prevalence stratified by different geographical areas in Iran

Figure 2 displays the crude prevalence data from the selected studies, categorized by geographical location, along with the pooled estimate derived from the meta-analysis. Eight articles published up to 2022 included in meta-analysis with a total of 369,103 diagnosed cases of alzheimer’s disease and dementia. The sample sizes of included studies ranged from 96 to 368,528.The overall prevalence was found to be 0.08 (95% CI; 0.07–0.09). Specifically, the prevalence in central and east region was estimated at 0.14 (95% CI; 0.04–0.31) which is very higher than west region.

**Fig. 3:**
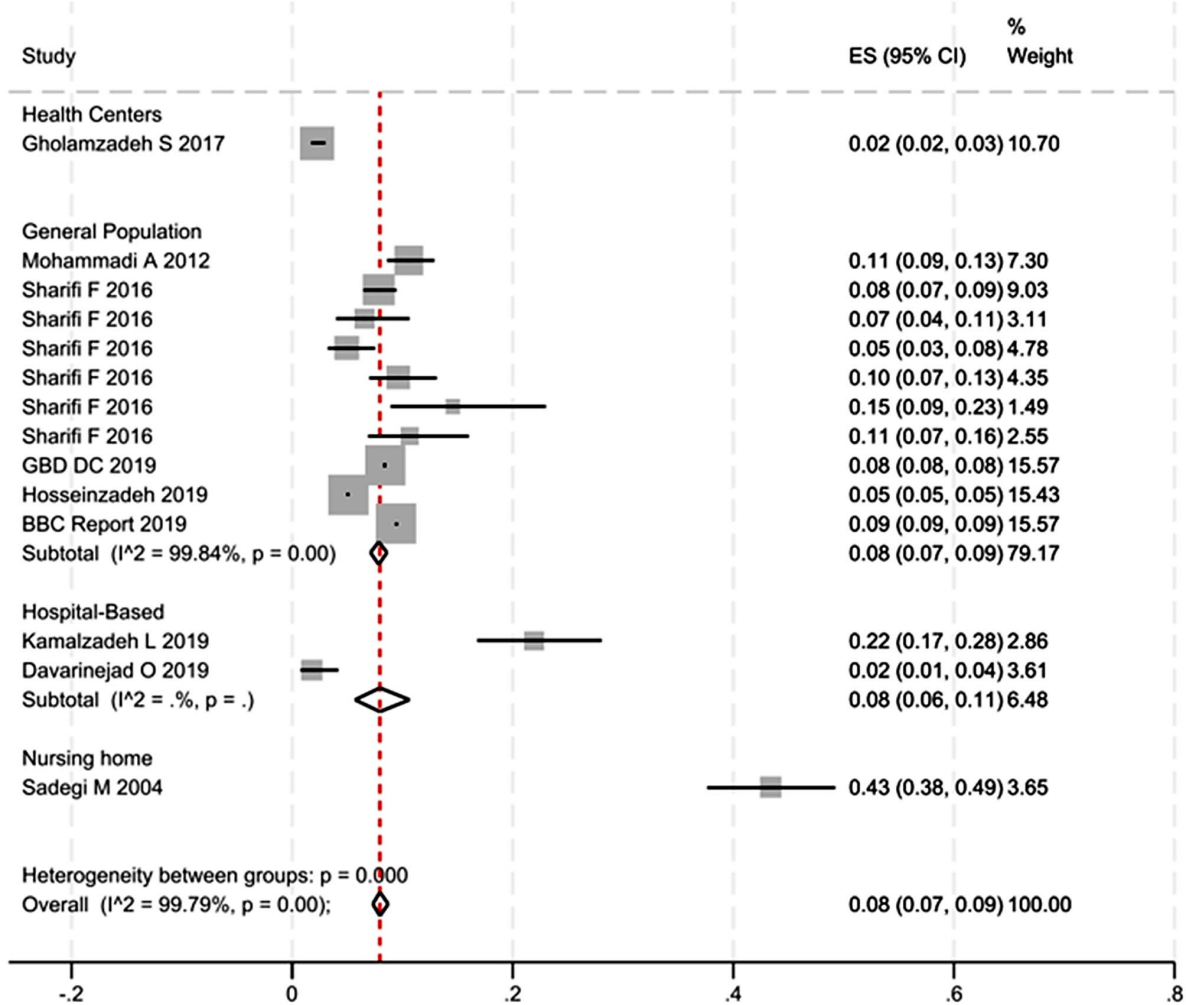
Estimated pooled prevalence of dementia by type of sampling

As illustraded in Fig. 3 prevalence estimates from studies conducted in central and eastern Iran exhibited greater variability than those from studies conducted in western Iran. In western Iran, the reported prevalence estimates were consistent with each other and demonstrated low overall variability. The study conducted in Kermanshah in nursing home, yielded results that deviated from those of other studies in this geographic region. This deviation is mainly due to a higher risk of dementia in this population.

In Fig. 3, the study conducted by Golamzadeh et al., which employed health centers sampling, yielded an estimated prevalence of 0.02 (95% CI; 0.02–0.03). Hospital-based studies, on the other hand, yielded an estimated prevalence of 0.08 (95% CI; 0.06–0.11). One study utilized a nursing home sampling method, resulting in an estimated prevalence of 0.43 (95% CI; 0.38–0.49) which is very high prevalence in compare to other studies and lead to high heterogeneity score of overall pooled prevalence. This study was also of low quality based on the NOS checklist used for quality assessment.

A study conducted in the west of Iran used a sampling method from a health center. The prevalence estimates from studies that sampled from the general population and were mostly of high quality were found to be consistent with each other. This indicates that the results obtained from these studies were reliable and accurate. The estimates obtained from two studies with hospital sampling were very different from each other. The prevalence estimates obtained from a study conducted in a nursing home in the west of Iran were found to be very different from other reported estimates.

In Fig. 3, high levels of heterogeneity (I^2^ > 75%, *P* > 0.01) were observed in the included studies due to both the sampling design and the geographical locations of the studies.

**Fig. 4:**
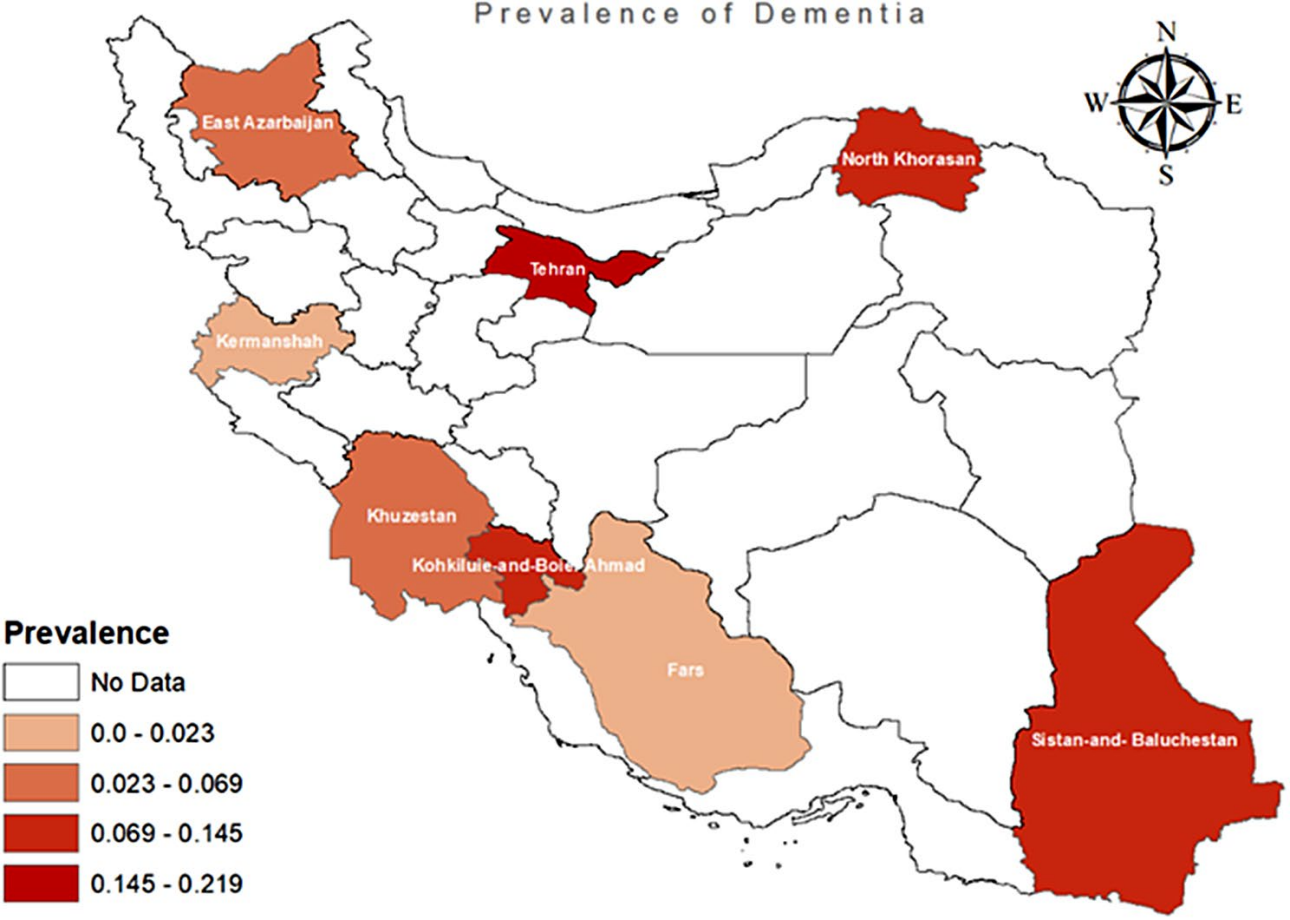
Spatial distribution of alzheimer prevalence in Iran using previous studies

Figure 4 illustrates the spatial distribution of Alzheimer’s prevalence in Iran based on the reported studies. It is not possible to determine the most studied provinces in Iran for dementia prevalence based on the available information. Therefore, the search results did not provide specific information to illustrate the country in the context of dementia prevalence research. Most dementia research focuses on marginal regions in Iran, compared to central provinces.The observed prevalence was high in Tehran as a capital city and comparatively low in Kermanshah as a western province.

In order to assess the impact of separate studies on the estimated prevalence, a sensitivity analysis was conducted. Each study was serially excluded, and the total prevalence was recalculated to assess the stability of the results. As shown in the table below (Table 2), the overall prevalence obtained from the combined studies demonstrates a high level of stability when any of the studies are removed. As it showed in the table the GBD DC 2019 and Hosseinzadeh 2019 studies have a higher effect on pooled prevalenc in compare to other studies.

**Table 2:** Sensitivity analysis for the effect of removing each study on overall estimation

Study omitted	Prevalence	95% Conf. Interval	
Gholamzadeh S 2017	0.08102436	0.07118463	0.09086408
Mohammadi A 2012	0.07810502	0.06830331	0.08790674
Kamalzadeh L 2019	0.07791566	0.06821048	0.08762085
Sharifi F 2016	0.07861254	0.06876259	0.0884625
Sharifi F 2016	0.07868567	0.0689486	0.08842275
Sharifi F 2016	0.07889648	0.06914385	0.08864911
Sharifi F 2016	0.07846612	0.06871334	0.08821891
Sharifi F 2016	0.07845814	0.06873794	0.08817834
Sharifi F 2016	0.07849464	0.06876254	0.08822674
Davarinejad O 2019	0.07901687	0.06928581	0.08874793
Sadegi M 2004	0.076338	0.06689396	0.08578205
GBD DC 2019	0.08993812	0.06228822	0.11758802
Hosseinzadeh 2019	0.08878209	0.07915617	0.098408
BBC Report 2019	0.08410058	0.05980253	0.10839864
Combined	0.07861584	0.06891338	0.0883183

## Discussion

The primary objective of the present study was to validate the pooled prevalence of alzheimer’s disease and dementia in Iran by conducting a systematic review and meta-analysis. Previous research on the epidemiology of alzheimer’s disease and associated risk factors in Iran has been limited, with only four selected studies encompassing a total sample size of 2781 participants. In this article, we included data from eight additional articles that have reported on the prevalence or frequency of dementia and Alzheimer’s disease, making them eligible for inclusion in our meta-analysis. According to a study conducted in 2017, the estimated prevalence of alzheimer’s disease among individuals aged 67–78 years old was found to be 2.3% [7]. In the most recent Iranian research were published in 2022, the indirect estimated prevalence of dementia was determined based on the frequency of specialized medication prescriptions within one year. The findings indicated a prevalence rate of 49.6 per 100,000 in the general population and 508.9 per 100,000 in individuals over 60 years of age [14].

In our study, we conducted an analysis of eight prior studies conducted in different provinces to estimate the overall crude prevalence of dementia. The results showed that the estimated prevalence was 0.08 (95% CI; 0.07–0.09). Additionally Studies that were based on a large and diverse sample of the general population were robust and effective in depicting dementia prevalence.

Furthermore, findings from the National Elderly Health Survey conducted in 2012, which included a sample from five provinces in Iran, indicated an overall crude prevalence of dementia at 7.9%, with rates of 8.7% in women and 6.9% in men. Moreover, when age-standardized, the prevalence of dementia in Iran was reported as 8.1%, with rates of 9.6% in women and 6.5% in men [15]. In earliest study using indirect estimating the prevalence of dementia in the 60-year populationutilized that were used the frequency of prescribed specialized medicines in 2015, resulting in an indirect estimated prevalence of 508.9 per 100,000 individuals [14].

Regarding regional variations, a study conducted in 2012 revealed that the overall diagnosis rate of dementia in Iran was 21.2%, with a range of 9.7–40% in Khuzestan and Alborz provinces. Although the diagnosis rate of dementia in Iran appears to be lower compared to developed countries, it is still more common than in many other developing countries [15]. Consistent with these findings, a study conducted in Europe between 1993 and 2008 reported an age- and sex-adjusted prevalence of dementia at 7.1% [8]. The estimated prevalence rates of dementia in neighboring countries were not available from the search results. However, a community study in Turkey, located northwest of Iran, revealed a dementia prevalence of 16.8% in Sivas city province. Moreover, statistics from the 2019 Global Burden of Disease (GBD) database indicate that Turkey, Bahrain, and Iran exhibit the highest age-standardized incidence rates (ASIRs) of dementia among the 204 countries and regions examined, while the lowest rates are observed in India, Pakistan, and Nepal [16].While the search results did not provide specific prevalence rates for dementia in neighboring countries, they did shed light on the prevalence of dementia in Iran and the elevated age-standardized incidence rates in Turkey, Bahrain, and Iran compared to other countries.

Between 1990 and 2018, the documented occurrence of dementia in Arab countries varied from 3.5 to 18.5% among individuals aged 80 years and above [17]. Another meta-analysis, encompassing multiple studies conducted across Asia, Africa, South America, and Europe/North America from 1985 to 2019, compiled data on the overall prevalence of dementia and Alzheimer’s disease. The pooled prevalence of all-cause dementia was calculated at 697 cases (95% CI: 546–864) per 10,000 individuals, while for Alzheimer’s disease it stood at 324 cases (95% CI: 228–460) per 10,000 individuals. Notably, the analysis highlighted a higher prevalence of these conditions among individuals aged 50 years and above, with women exhibiting greater susceptibility compared to men (788 cases versus 561 cases per 10,000 individuals) [18].

A scarcity of comprehensive studies has direct estimations of dementia prevalence in other Middle East nations. Limited data and underreporting, as well as varying incidence and risk profiles, contribute to this scarcity [19]. Furthermore, access to information on the cumulative incidence of dementia is restricted in several Middle East countries, suggesting a lack of awareness in the region. The risk of dementia is reported to be influenced by a diverse range of factors, and the region is considered relatively young, with dementia unlikely to become an epidemic or pose a looming threat in the Middle East and North Africa [20]. In a recent study, it was estimated that there were 55 million people living with dementia globally in 2021, and this number is projected to rise to 78 million by the end of 2030 [21]. These comparisons, which highlight the growing prevalence of dementia in Middle East countries, including Iran, demonstrate the need for further research to accurately assess the situation. The region faces challenges such as under-reporting, a lack of knowledge, and limited resources for dementia treatment.

The pooled prevalence of dementia and alzheimer’s disease among individuals aged 55 years and older in China was estimated to be 4.03% and 2.44%, respectively, from 1985 to 2015 [22]. Additionally, the Toyama Dementia Survey in Japan, which faces challenges due to its aging population, projected that the estimated future prevalence of dementia in 2025 will exceed 20% in predominantly rural areas [23].Today these Asian countries are grappling with the challenges of an aging population, such as dementia. As reported above, dementia prevalence in Iran is lower than other Asian countries, that could be attributed to yongerness of Iran’s population than others (like Japan and China). These countries should implement policies that capitalize on their demographic shifts to address the needs of their aging populations.

Nonetheless, the estimation of prevalence for dementia and alzheimer’s disease can be significantly influenced by factors such as the diverse diagnostic criteria utilized and the aptitude of physicians in diagnosing these conditions. Additionally, the subset of individuals included in the sampling process further contributes to variations in prevalence estimates. In our particular study, we acknowledge the substantial impact of the sampled population subset on the overall prevalence assessments. As a result, we have reported three distinct categories of prevalence based on different populations that were sampled: the general population (as documented in 5 articles and documents), health centers (as indicated in 1 article), hospital-based populations (as described in 2 articles), and nursing homes (as detailed in 1 article).

Our results estimated prevalence of dementia in Iran ranges from 0.02 to 0.43%. This high variation mainly due to location of sampling. It’s essential for recall, the estimate of dementia prevalence can be significantly affected by sampling methods, especially when comparing the general population to those used in health facilities or hospitals. Research shows that estimates derived from the general population are different from estimates derived from certain groups, such as patients seeking treatment at hospitals or health centers [24–26]. While health center or hospital-based sampling can provide valuable dementia prevalence data, it is essential to consider the potential biases that can lead to overestimation or underestimation, such as selection bias and limited generalizability [27–28]. On the other hand, community-based sampling, such as random sampling, can offer a more representative sample of the general population but may face limitations, including underrepresentation of specific groups, challenges in accessing certain populations, variability in diagnostic assessment, and resource intensity [15]. These limitations can hinder the feasibility of conducting large-scale prevalence studies, particularly in low-resource settings [29].

Our findings indicate that in Iran, dementia prevalence varies by geographical region. In the Central and East provinces, the prevalence was 0.14 (95% CI: 0.04–0.31), while in the West provinces it was 0.06 (95% CI: 0.03–0.11). Another study conducted in 2012, which involved a national survey, revealed that East Azerbaijan had the lowest age-sex adjusted prevalence of dementia, whereas North Khorasan had the highest [15]. Furthermore, by indirectly estimating disease prevalence based on the frequency of prescribed specialized medications in 2015, it was determined that Hormozgan Province had the lowest prevalence rate (9.4/100,000), while East Azarbayjan Province had the highest (96.4/100,000). Among the population aged over 60 years, the lowest prevalence was observed in Hormozgan Province (141.5/100,000), whereas the highest prevalence was recorded in Isfahan Province (862.5/1,000,000) [14]. These results align with our own findings, indicating a higher prevalence of the disease in the central regions of Iran and a lower prevalence in the southeastern and western parts of the country Additionally It’s crucial to remember that getting indirect estimates of dementia prevalence might be challenging due to differences in research methods and diagnostic criteria. Yet their results are vital because they provide important information on the prevalence of dementia in all provinces.

Beyond the points mentioned, further consideration is warranted regarding the geographical distribution of dementia in Iran. Numerous factors have been identified as contributing to the prevalence of dementia in Iran, including age, genetics, depression, hypertension, dietary patterns, and educational level. A systematic review of the literature estimated the prevalence of Alzheimer’s disease in Iranians aged 67–78 years 2.3%, attributing this to age, genetics, depression, and hypertension [7]. A case-control study revealed a significant association between a history of vitamin D deficiency, cancer, and chronic anemia and an increased risk of dementia [30]. The National Elderly Health Survey in Iran identified diabetes mellitus, depressed mood, illiteracy, and advanced age as significant risk factors for dementia [15]. Moreover, the Iranian Registry of Alzheimer’s disease projected an increase in the number of individuals with dementia due to rising life expectancy and an expanding elderly population [31]. The variation of these risk factors across Iran’s geographical regions may further contribute to the prevalence of dementia, underscoring the need for comprehensive strategies to address this growing public health concern.

Despite the limited number of articles reviewed, the findings of the current systematic review are reasonably sound. It is evident that Iran is grappling with an increasing burden of dementia due to its aging population. While there has been limited research focusing on dementia and alzheimer’s at the population level in Iran thus far, other healthcare initiatives, such as the aging health program, present an excellent opportunity to enhance alzheimer’s disease research through data linkage processes. Limited public awareness of dementia and alzheimer’s disease persists in Iran, leading to misattribution of early symptoms to normal aging.

The caregiving of alzheimer’s disease patients in Iranian society is predominantly provided within their homes due to familial dependence. The implementation of principles for domestic healthcare for elderly Iranians with dementia can notably improve the overall well-being of these patients in their home environment [32]. Further analysis is warranted to devise effective strategies for enhancing awareness of dementia and its associated risks.

Drawing from the expertise gained through the Tehran registry of alzheimer’s disease, establishing population-based registry centers in other cities of Iran could furnish valuable datasets to strengthen the research infrastructure, similar article has been done in Australia which introduced registry for dementia and and mild cognitive impairment [33]. Furthermore, this article emphasizes the development and design of a community-based Alzheimer’s Registry, which would contribute to broadening the diversity of research samples, By doing so, a more accurate numerator and denominator for calculating prevalence ratios can be obtained compared to other sampling methods commonly employed in alzheimer’s research.

In conclusion, this is the first systematic review and metaanalyses on the prevalence of dementia in Iran considering geographic distribution of the disease in the country using published studies. Additionally, it was found that the prevalence of dementia in Iran was lower than in other Asian countries, which could be attributed to the relatively younger population in Iran than others. As mentioned in the study, most dementia research was conducted in marginal provinces, compared to central provinces. This highlights the need to conducting dementia prevalence research in these provinces to better illustrate the status of dementia in Iran.

### Limitation 

The studies on the prevalence of dementia in Iran were limited to marginal provinces leasd to selection bias, less representative estimated prevalence. Moreover, in the division of studies based on the set of sampling, the confidence interval of some estimates is wide due to random error, which can contribute to imprecise estimates.

### Electronic supplementary material

Below is the link to the electronic supplementary material.

Supplementary Material 1

## Data Availability

Data is provided within the supplementary information files.
